# Apoptosis Induction of Fibroblast-Like Synoviocytes Is an Important Molecular-Mechanism for Herbal Medicine along with its Active Components in Treating Rheumatoid Arthritis

**DOI:** 10.3390/biom9120795

**Published:** 2019-11-28

**Authors:** Qing Zhang, Jia Liu, Mengmeng Zhang, Shujun Wei, Ruolan Li, Yongxiang Gao, Wei Peng, Chunjie Wu

**Affiliations:** 1 School of Pharmacy, Chengdu University of Traditional Chinese Medicine, Chengdu 611137, China; zq1995729@163.com (Q.Z.); liujia1997free@163.com (J.L.); garita119@163.com (M.Z.); 734695116@qq.com (R.L.); 2 School of Basic Medicine, Chengdu University of Traditional Chinese Medicine, Chengdu 611137, China; ShujunWei05@163.com (S.W.); gaoyxcdtcm@126.com (Y.G.)

**Keywords:** apoptosis, signal pathway, herbal medicine, fibroblast-like synoviocytes, rheumatoid arthritis

## Abstract

Rheumatoid arthritis (RA) is a known chronic autoimmune disease can cause joint deformity and even loss of joint function. Fibroblast-like synoviocytes (FLS), one of the main cell types in synovial tissues of RA patients, are key effector cells in the development of RA and are considered as promising therapeutic targets for treating RA. Herbal medicines are precious resources for finding novel agents for treating various diseases including RA. It is reported that induction of apoptosis in FLS is an important mechanism for the herbal medicines to treat RA. Consequently, this paper reviewed the current available references on pro-apoptotic effects of herbal medicines on FLS and summarized the related possible signal pathways. Taken together, the main related signal pathways are concluded as death receptors mediated apoptotic pathway, mitochondrial dependent apoptotic pathway, NF-κB mediated apoptotic pathways, mitogen-activated protein kinase (MAPK) mediated apoptotic pathway, endoplasmic reticulum stress (ERS) mediated apoptotic pathway, PI3K-Akt mediated apoptotic pathway, and other reported pathways such as janus kinase/signal transducers and activators of transcription (JAK-STAT) signal pathway. Understanding the apoptosis induction pathways in FLS of these herbal medicines will not only help clear molecular mechanisms of herbal medicines for treating RA but also be beneficial for finding novel candidate therapeutic drugs from natural herbal medicines. Thus, we expect the present review will highlight the importance of herbal medicines and its components for treating RA via induction of apoptosis in FLS, and provide some directions for the future development of these mentioned herbal medicines as anti-RA drugs in clinical.

## 1. Introduction

Rheumatoid arthritis (RA) is a chronic, invasive autoimmune disease that can cause joint deformity, even complete loss of joint function, so it is often referred to as “Deathless cancer” [1]. Pathological characteristic of RA is excessive synovial tissue hyperplasia, pannus formation, and erosion of cartilage, and the typical clinical features is the symmetry of joint swelling, pain, morning stiffness, and deformity, often accompanied by joint organ involvement [2,3]. RA can occur at any age, with the highest incidence occurring between the ages of 40 and 60 years, and it is more common in women, and the incidence is about four times that of men [4,5]. In China, the prevalence rate of RA is 0.2–0.4%, while in European and American countries, the prevalence rate of RA can be as high as 1% [6,7,8]. At present, the clear pathogenesis of RA is still uncovered, this immune disease has a high disability rate, poor prognosis, and is prone to repeated attacks. Meanwhile, the treatment cycle is very long, so it will bring heavy economic burden to patients’ families and society. Currently, the treatment strategies for RA in clinical practice are mainly chemical drugs, such as non-steroidal anti-inflammatory drugs (NSAIDs), disease-modifying anti-rheumatic drugs (DMARDs), glucocorticoids, and biological agents [1,8]. However, these treatments are costly and often associated with adverse reactions, such as cardiovascular and gastrointestinal bleeding risk, liver and kidney toxicity, growth inhibition, infection, and tumor risk [9,10,11,12].

With the number of RA patients increasing year by year, medicinal researchers are actively looking for cheap and effective alternative drugs with fewer side effects to treat RA. Recently, herbal medicines have been given more and more attention for their remarkable curative effects and fewer side effects [13,14]. Herbal medicines have been used for the clinical management of RA for thousands of years, and its efficacy and safety have been proved by its long-term clinical application [15,16]. It is interesting and known that herbal medicines can through multiple components act in multiple pathways to give play to the role of prevention and treatment of RA. The main components in herbal medicines for treating RA are complex including alkaloids, flavonoids, terpenes, phenylpropanins, etc., and the main pharmacological effects are related to pain relief, improvement of inflammation, regulation of immune function, protection of cartilage, reduction of pannus formation, inhibition of synovial hyperplasia, etc. [16,17]. Fibroblast-like synoviocytes (FLS), one of the main cell types in the synovial tissues of RA patients, are key effector cells in the development of RA, and are considered as promising therapeutic targets for treating RA [1,18]. It is worth noting that a large number of herbal medicines and their monomers seem to have a good effect for inhibiting synovial hyperplasia of arthritis, and the detailed molecular mechanisms are mainly related to inhibiting proliferation of FLS via induction of apoptosis. However, there is no systematic analysis or summary regarding the apoptotic effects of herbal medicines on FLS for treating RA. Thus, our study aims to review the important researches on herbal medicines and its monomer components inducing FLS apoptosis, and summarize the related possible molecular signal pathways for apoptosis, so as to provide references for the follow-up studies of herbal medicines on treating RA.

## 2. Cell Apoptosis

Currently, the cell death modes discovered by humans mainly include apoptosis, necrosis, autophagy, and pyroptosis, which have great differences in morphological characteristics and biochemical signal transduction [19]. Apoptosis is the cell independent orderly death in order to resist the external stimulation and maintain the homeostasis of the internal environment, which is often referred to as programmed death. Different from other ways of cell death, apoptosis is not a self-injury phenomenon, but a self-protection mechanism, which is activated, expressed and regulated by a series of specific genes [19,20]. In 1972, Kerr et al. first proposed the term apoptosis to describe a morphological feature of cell death that had never occurred before [21]. Programmed cell death during the development of *Caenorhabditis elegans* has led to the recognition and understanding of the mechanisms involved in mammalian apoptosis [22]. Apoptosis is a basic physiological phenomenon, which plays an important role in organism growth, development, and evolution [23]. On the one hand, apoptosis can maintain the homeostasis and the dynamic balance of cell number in the body, and on the other hand, it can be used as a defense mechanism to eliminate unnecessary or abnormal cells [24]. Appearing in cell apoptosis, cell shrinkage, smaller volume, nuclear enrichment, nuclear membrane nucleoli, DNA fragmentation, then the cell cleaves into apoptotic corpuscles, which are formed by the cell membrane enclosed cytoplasm, organelles and broken nucleus, and eventually the apoptotic body are recognized around the macrophage, which in turn being swallowed, degradation. During the whole process of cell apoptosis, the cell membrane structure is complete, no contents are spilled, no cytokines are released, and the duration is short, so the surrounding inflammatory reaction is basically not caused. However, when abnormal regulation of this cellular program occurs in the body, it can induce many serious diseases, such as tumors, cardiovascular diseases, autoimmune diseases, etc. [25].

## 3. Apoptosis of FLS in RA Patients and RA Pathology

RA is a complex disease due to heterogeneous reasons, and the precise etiology of this disease remains unclear until now. Increasing scientific evidence has suggested that FLS, also named synovial lining fibroblasts, play a key role in the development of RA [18,26,27]. For the pathogenesis of RA, characteristic of RA is excessive synovial tissue hyperplasia, pannus formation, and erosion of cartilage, and the excessive proliferation and inadequate apoptosis of FLS is generally recognized as the pathological basis of RA.

Normal synovial fibroblasts are mainly distributed in synovial lining layer, secreting appropriate synovial fluid to reduce bone friction and reduce joint injury, and secreting various cytokines to nourish joints and ensure the normal progress of joint activities. When synovial fibroblasts have stable activation, the defects in apoptosis, began with abnormal proliferation and transformation in tumor samples, showed similar characteristics of aggression and adhesion [7,28]. It is reported that FLS in RA patients are resistant to apoptosis due to the unbalance of the anti- and pro-apoptotic molecules, and the increasing evidences have revealed that anti-apoptotic mediators, such as Bcl-2, Mcl-2, and FLICE-inhibitory protein (FLIP), are up-regulated in the FLS of RA patients whereas the pro-apoptotic proteins, such as tumor necrosis factor-related apoptosis-inducing ligand (TRAIL), p53 up-regulated modulator of apoptosis (PUMA) and Bid, are down-regulated in the FLS of RA patients [29,30,31,32]. In addition, it is also reported that increased p53 mutants are found in the FLS of RA patients (RA-FLS) whereas the p53 expressions were relatively decreased, which is considered one of the important reasons for the excessive proliferation and inadequate apoptosis of FLS [31]. In addition, RA-FLS increase the expression of matrix metalloproteinases (MMPs), degradation of cartilage extracellular matrix (ECM), blocking of articular cartilage nutrition supply, infiltration and organization of the joint destruction [18]. On the other hand, FLS secretes a variety of chemokines, such as RANTES, IP-10, ENA-78, MCP-1, SDF-1, CXCL16, etc., which can recruit macrophages, T cells, and B cells to migrate to joints, enhance inflammatory response, and indirectly destroy bones and joints. In addition, FLS can also inhibit the apoptosis of T and B cells, so that the inflammatory response persists, reinforcing the damage to bones and joints [33]. In summary, the apoptotic defect of FLS can lead to abnormal synovial hyperplasia, pannus formation, and inflammatory cell infiltration, resulting in cartilage and bone erosion, joint destruction, joint deformity, and eventually joint function loss. Therefore, how to effectively promote the apoptosis of FLS and inhibit synovial hyperplasia has important clinical significance for the treatment of RA, providing a feasible direction for the development of anti-RA drugs.

## 4. Effects and Mechanisms for Herbal Medicine and its Components on FLS Apoptosis in RA

### 4.1. Death Receptors Mediated Apoptotic Pathway

Death receptors mediated apoptosis, known as the extrinsic apoptotic pathway, is one of the main well-characterized apoptotic routes [34]. The death receptor mediated apoptotic pathway is mainly triggered by extracellular stimuli which are commonly recognized by the tumor necrosis factor receptor (TNFR) family of proteins (also called death receptors), such as the TNFR, Fas, and TRAIL-R [20,35,36]. Activated death receptors by their ligands, such as TNF-α, FasL, and TRAIL, would further form the death inducing signaling complex (DISC), and subsequently bind to the Caspase-8, resulting in the activation of Caspase-8 by dimerization. Then, the activated Caspase-8 could further activate the Caspase-dependent apoptotic pathway via two routes [20]. Firstly, the activated Caspase-8 catalyze and active the executioner Caspases (including Caspase-3, -6, and -7), lead to the apoptosis. Secondly, the activated Caspase-8 could trigger the intrinsic mitochondrial pathway via the Bid cleavage, following a series of apoptotic events, such as cytchrome c (Cyt-C) release from the mitochondria, apoptosome formation, Caspase cascade reaction, and PARP cleavage, and eventually lead to apoptosis [20,37,38,39]. Currently, increasing evidences have suggested that induction of apoptosis in FLS via death receptors mediated apoptotic pathway is an important molecular mechanism for herbal medicine extracts and its active components to treatment of rheumatoid arthritis.

#### 4.1.1. Herbal Medicine Extracts

In 2002, a study reported by Liu et al. revealed that a clinical RA drug of *Xinfeng* capsule (XFC, 1.8 g/kg, p.o.) possessed significant anti-RA effects in rats via reduction of arthritis index, and the potential mechanisms are correlated to up-regulation of Fas and FasL, whereas down-regulation of Bcl-2 in synovia tissues of joints [40]. Furthermore, Liu et al. (2010) investigated the effects of extracts from the roots of *Salvia miltiorrhiza* (ERSM) on FLS of clinical RA patients (RA-FLS). From the results of this investigation, Liu et al. suggested that ERSM (0.4 mg/mL) has obvious apoptosis inducing activities in RA-FLS via up-regulation of mRNA expressions of Fas [41]. In addition, another research article by Liang et al., in 2017, investigated the anti-RA effects of an interesting traditional Chinese medicine (TCM) clinical prescription, named *Fengshi Bitong* Prescription (FSBT), and reported that FSBT (9.5, 19, and 38 g/kg, p.o.) could decrease the paw swelling of collagen-induced arthritis (CIA) rats. Similar to tripterygium glycosides, the potential molecular mechanisms of FSBT are closely correlated to the regulation of Caspase-8, Fas and FasL in synovial tissues of CIA rats [42].

#### 4.1.2. Monomers from Herbal Medicine

**Figure 1:**
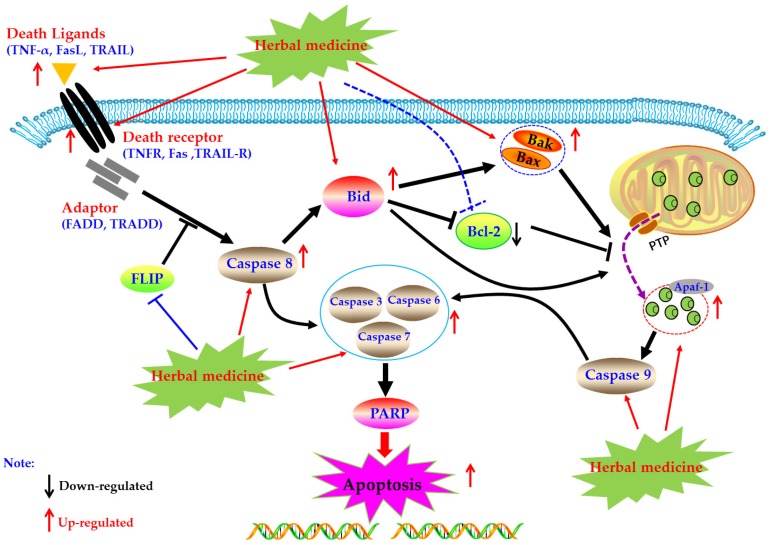
Death receptors mediated apoptotic pathway in FLS induced by herbal medicines.

In 2008, Zhao et al. reported that Resveratrol (**1**), which is a natural active compound existed in *Polygounm cuspidatum* [43], has promising curative effects on RA symptoms of CIA rats and could induce the primary cultured FLS (rFLS) from CIA rats via up-regulation of Caspase-8, whereas down-regulation of FLICE inhibitory protein (FLIP) [44] which is an important anti-apoptotic protein that can suppresses the death receptors ligands (such as TNF-α, FasL, and TRAIL) induced apoptosis in FLS of RA patients (RA-FLS) (Figure 1), and is also an important reason for the resistance of RA-FLS to apoptosis [45,46,47]. Later, in 2013, it is reported that Propyl gallate (**2**) (64 μg/mL) which is an active component in Radix paeoniae rubra could induce the apoptosis of RA-FLS via up-regulating the mRNA expressions of Fas [48,49]. Furthermore, a study reported that Daphnetin (**3**) (40 μg/mL) significantly induced apoptosis in rFLS from CIA rats, and the related molecular mechanisms were closely related to up-regulation of Caspase -3, -8, and -9 and FasL [50,51]. The potential mechanisms of herbal medicine for inducing apoptosis in this part are summarized in Figure 1 and Table 1.

### 4.2. Mitochondrial Dependent Apoptotic Pathway

**Figure 2:**
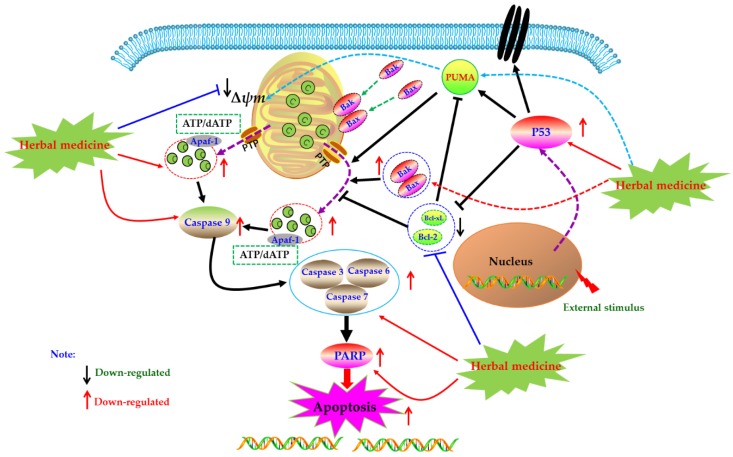
Mitochondrial dependent apoptotic pathway in fibroblast-like synoviocytes (FLS) induced by herbal medicines.

Mitochondrion, as an important organelle of cell, is the energy factory of cell activities. The integrity of its structure and function is an important premise to ensure the normal life activities of cells. Mitochondria mediated cell apoptosis is also known as the endogenous pathway of cell apoptosis, which is one of the important ways of cell apoptosis. In 1994, Newmeyer et al. reported that mitochondria in the extract of *Xenopus afroensis* egg could coagulate the nucleus chromatin, and the nucleus showed contractile and fragmented, suggesting that mitochondria were closely correlated to programmed cell death [52]. Since then, increasing studies have also proved that mitochondria play an important role in the process of cell apoptosis, which is the key element of apoptosis. Briefly, when cells receive apoptosis signals from p53-PUMA signal or death receptor signal pathways (Figure 2), the pro-apoptotic proteins of Bcl-2 family were up-regulated and activated, whereas expressions of anti-apoptotic proteins were down-regulated. The pro-apoptotic proteins, such as Bax and Bak, were transferred from the cytoplasm to the mitochondrial membrane, forming transmembrane pores and decreasing the mitochondrial membrane potential (MCMP). Meanwhile, due to breaking of the balance of pro-/anti-apoptotic proteins, the permeability transition pore (PT) was induced to open, which further reduced the mitochondrial membrane potential (MCMP), increased the permeability of the mitochondrial membrane, resulting in the releases of cytochrome C (Cyto C). With the participation of ATP or dATP, Cyto C enters the cytoplasm and subsequently binds to Apaf-1 and other apoptotic protease activators to form apoptotic complexes. Apoptosis complexes recruit and activate the initiator cysteinyl aspartate specific proteinase (Caspase), such as Caspase 9, etc. Then, the activated initiators activate executioner Caspases (such as Caspase-3, -6, -7), and start the Caspase cascade, cutting poly ADP-ribose polymerase (PARP), actin and other substrates, eventually leading to the cell death and cell lysis [20,26,27]. As natural products, herbal medicines and its active ingredients are considered as effective strategies for RA treatment due to their good efficacy and low toxicity. Meanwhile, increasing studies have proved that these drugs can play anti-RA effects by mediating mitochondrial apoptosis pathway in FLS.

#### 4.2.1. Herbal Medicine Extracts

An investigation in 2011 by Dai et al. studied the anti-RA effects of seaweed polysaccharide (SWPD) in vitro on RA-FLS, and the results revealed SWPD (15, 20, 25 mg/mL) has significantly anti-proliferative activity on RA-FLS via inducing mitochondrial apoptosis pathway in RA-FLS through up-regulating Bax and Caspase-3 whereas down-regulating Bcl-2 [53]. Later in 2013, it is also reported that an Chinese patent drug called *Feng-shi-ning* capsule has significant anti-rheumatic activities and the related mechanisms were related to induction of mitochondrial dependent apoptosis in via increasing the releases of Cyt-C, up-regulating Caspase-3, as well as down-regulating Bcl-2 [54]. In 2018, Gao and Lu studied effect of medicated serum of *Duhuo Jisheng* decoction (DHJS medicated serum, 0.75, 1.5 and 3 g/kg) on rFLS from Freund’s adjuvant-induced arthritis (AIA) rats, the results showed that 20% DHJS medicated serum can induce apoptosis in rFLS (AIA), and the mechanisms are related to regulating Bax, Bcl-2 and Caspase-3 [55]. Furthermore, a research by Ci et al. isolated an interesting polysaccharide from the *Saposhnikovia divaricate* (SDP), and the SDP (5–15 mg/mL) dose-dependently induced apoptosis in the rFLS from AIA rats. The possible mechanisms are closely related to up-regulating p53, Bax and Caspase-3, whereas down-regulating Bcl-2 [56]. Later in 2019, Wu et al. investigated the apoptosis inducing effects of Pterocarya Hupehensis Skan extracts (PHSE) on MH7A cells, and the results showed that PHSE (25, 50, 100 μg/mL) resulted in obvious anti-proliferative and apoptotic effects in MH7A cells. The further mechanisms research revealed that PHSE can up-regulate p53, Bak, Cyt-C, Caspase-3, -9 and Bax, whereas down-regulate Bcl-2 and Bcl-xL, and activate the Caspase-3 in MH7A cells [57]. Recently, Zhang et al. investigated the curative effects of *Guizhishaoyaozhimu* Decoction (GZSD), which is a known classical prescription of traditional Chinese medicine (TCM), on type II collagen-induced arthritis (CIA) in rats. It is found that GZSD has a significant anti-arthritic effect on CIA rats, and the further in vitro study in MH7A cells revealed GZSD (0.4, 0.8, 1.6 mg/mL) could induce apoptosis in MH7A cells and up-regulate pro-apoptotic proteins such as Caspase-3, -9, Bax and down-regulate Bcl-2 [16].

#### 4.2.2. Monomers from Herbal Medicine

In the year of 2009, Li et al. reported that after 24 h treatment with Scopoletin (**4**) (250, 500, 1000 μM), the MCMP (Δ*ψ*m) of FLS in AIA rat was depolarized, resulting in mitochondrial dependent apoptosis; and the further studies found that Scopoletin can activate caspase-3 and down-regulate Bcl-2 whereas up-regulate Bax [58]. Later in 2010, Li et al. found that the 7, 3′-Dimethoxy hesperetin (**5**) (DMHP, 10, 50, 250 μM), a derivative of hesperidin, can significantly decrease the proliferation of the rFLS from AIA rats via induction of apoptosis, which is closely related to regulation of Bax, Caspase-3 and Bcl-2, and activation of Caspase-3 in rFLS (AIA) [59]. Another work in 2011 by Wang reported that Berberine (**6**) (5–75 μM) can induce apoptosis in RA-FLS by increasing Caspase-3, -9, Bax and PARP, whereas decreasing MCMP (Δ*ψ*m), Bcl-2 and Bcl-xL [60]. Later in 2012, after prepared the primary cultured rFLS from AIA rats, Ren et al. investigated effect of the 5,7,3′-Triacetyl hesperetin (TAHP, **7**) on rFLS (AIA), and they found that TAHP (50 and 250 μM) can notable inhibit the proliferation of rFLS (AIA) in vitro, and the molecular mechanisms might be related to induction of apoptosis via up-regulating Bax, Caspase-3 whereas down-regulating Bcl-2 in rFLS (AIA) [61]. In another study by Yan et al. in 2012, Andrographolide (**8**) (10, 20 and 30 μM) showed a highly anti-rheumatic activity on RA-FLS from RA patients cultured in vitro via inducing apoptosis, which was associated with decrease of the Bcl-2/Bax ratio, promotion of Cyt-C release, and activation of Caspase-3 [62]. Moreover, a natural polyphenolic acid named Gallic acid (**9**) was investigated by Yoon et al. in 2013, and Yoon et al. found that this Gallic acid (0.1, 1 μM) could induce apoptosis in RA-FLS via regulation of Bcl-2, Bax, p53 and Caspase-3 [63]. Another work in 2013 by Xu suggested that Celastrol (**10**) (1, 2, 5 μM) also induced apoptosis in RA-FLS via increasing Caspase-3, -9, PARP, Fas and Bax, whereas decreasing Bcl-2 and MCMP (Δ*ψ*m) [64]. Besides, in 2013, another important natural monomer named Quercetin (**11**) (100–300 μM) also reported to be an effective agent can promote apoptosis in RA-FLS through regulation of Caspase-3, -9, Cyto C and Bcl-2 [65]. In 2014, Chang et al. reported that Bufalin (**12**) (10, 20, 40 nM), the major active digoxin-like component of Chansu isolated from the skin and parotid venom glands of toad, possessed significant anti-proliferative and apoptosis inducing activities on RA-FLS; the investigators found that Bufalin can up-regulate Bax, down-regulate Bcl-2, activate Caspase -3 and PARP, increase Cyt-C release, and decrease MCMP (Δψm), and consequently the authors thought mitochondrial mediated apoptosis might be one of the possible molecular mechanisms [66]. Additionally, another report by Jie et al. studied the effect of Tanshinone IIA (**13**), and found that this compound (2.5–20 μM) could induce apoptosis in RA-FLS by mitochondrial pathway via up-regulating Bax, cytosolic Cyto C, Apaf-1, Caspase-3, -9 whereas down-regulating Bcl-2 [67]. Afterwards, Liu et al. in 2016 recorded another natural coumarin constituent named Daphnetin (**3**) (40 μg/mL) extracted from *Daphne odora* could induce apoptosis in RA-FLS of CIA rats, and after Daphnetin treatment, the Caspase-3, -8, -9, Bax, Bid, Cyt-C were significantly up-regulated whereas the Bcl-2 were down-regulated [51]. In the study of Shang et al. (2016), it was found that Oridonin (**14**) (5, 10, 25, 40 μM) can induce the apoptosis in RA-FLS, regulate the mRNA expressions of Bax and Bcl-2, reduce cells’ MCMP, promote the outflow of Cyt-C, and activate Caspase-3 [68]. Besides, an in vitro study has found that Resveratrol (**1**) (50, 100, 200, 400 μM) can decrease the MCMP (Δψm), destroy the mitochondrial structure and function, trigger mitochondria mediated apoptosis in rFLS (AIA) [69]. Another report by Gu and Jin reported that Resveratrol (100, 200 μM) (**1**) inhibited the proliferation of RA-FLS via increasing the Bax expression while decreasing the Bcl-2 expression [70]. PUMA is a key protein that mediates p53-dependent and p53-independent apoptosis, and current investigations also found that natural monomers, such as Resveratrol and Gallic acid, can increase the expressions of p53 and PUMA to promote apoptosis of various cancer cells or fibroblasts [51,71,72]; thus we speculated that up-regulation of PUMA is also a possible target for herbal medicines and its constituents for treating RA. In 2016, Gao reported that Pristimerin (**15**) (0.4, 0.8 mg/kg, i.p.) significantly improved the RA symptoms of AIA rats, and further investigations revealed that Pristimerin (0.75–3 μM) can induce apoptosis of rFLS (AIA) via regulation of expressions of Bcl-2, Bax and Caspase-3 [73]. Liquiritin (**16**) is a known active constituent isolated from *Glycyrrhiza uralensis*, and recently, Zhai et al. reported that Liquirtin (0.345–34.5 μM) induced apoptosis in RA-FLS via down-regulating the ratio of Bcl-2/Bax [74]. Besides, it is reported that Cryptotanshinone (**17**) (5 μM), a fat soluble anthraquinone derivative isolated from the *Salvia miltiorrhiza*, could induce the apoptosis in MH7A cells and RA-LFS cells via reactive oxygen species (ROS) induced apoptosis by regulation of Bcl-2, Bad, cleaved-Caspase-3 and PARP [75]. The potential mechanisms of herbal medicine for inducing apoptosis in this part are summarized in Figure 2 and Table 1.

### 4.3. NF-κB Mediated Apoptotic Pathways

Under normal physiological conditions, cells receive extracellular signal stimulation, and receptor proteins on the membrane polyaggregate and transmit the signal to IKK (IκB kinase). IKK, a kinase, phosphorylates IκB, which is subsequently dissociated from the trimer complex formed with Nuclear factor-kappa B (NF-κB) [76]. The released NF-κB regenerates its activity, quickly enters the nucleus from the cytoplasm, and combines with specific sequences on DNA in the nucleus to control protein transcription, and participate in physiological processes such as cell proliferation and apoptosis, stress response, and release of cytokines. However, when NF-κB is abnormally activated, the body can develop a series of serious diseases, such as cancer, atherosclerosis, and rheumatoid arthritis. Beg et al. showed that fetal mice with defects in the Re1A subunit of NF-κB would show programmed cell death or apoptosis in the second trimester, leading to large-scale degradation of the liver and ultimately the death of the embryo. It was the first time that NF-κB was involved in the process of cell apoptosis. Subsequently, more and more studies have found that NF-κB is closely related to apoptosis [77].

**Figure 3:**
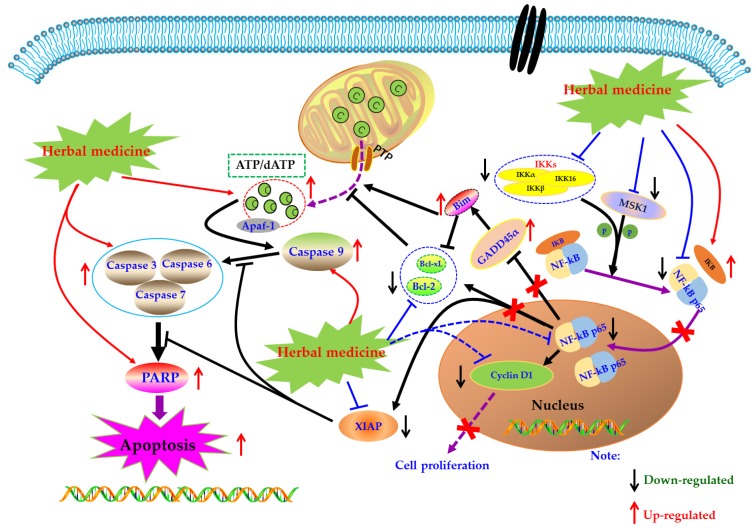
Mitochondrial dependent apoptotic pathway in FLS induced by herbal medicines.

Currently, NF-κB has attracted more and more attention as a potential therapeutic target in the management of RA [78,79,80]. Modern pharmacological studies have proved that natural products can induce FLS apoptosis to prevent synovial hyperplasia via regulation of NF-κB pathway. As early as 2007, Zhang et al. found that Sinomenine (SIN, **18**) (3.2 mM), an active alkaloid from the *Caulis Sinomenii*, can induce apoptosis in RA-FLS using flow cytometry analysis [81]. Later, in 2008, Fang et al. revealed the SIN (2.8–3.2 mM) resulted in significant cell cycle arrest and down-regulation of Bcl-2 in RA-FLS [82]. Another investigation in 2015 by Zhang et al. studied the effect of SIN on proliferation of RA-FLS, and found that SIN has significant anti-proliferative activity on RA-FLS via inhibition of NF-κB and mitogen-activated protein kinase (MAPK) signal pathway through down-regulating MyD88 and TRAF-6 proteins in RA-FLS [83]. In 2009, it is reported by Li et al. that besides mitochondrial dependent apoptosis, the anti-proliferative effects of Scopoletin (**4**) on RA-FLS also closely related to NF-κB pathway via regulation of Bax, IκBα, p-IKK and p-IκBα [57]. Later, in 2010, Shang et al. reported that Curcumin (**19**) (80 μM) could inhibit the proliferation and arrest the cell-cycle of RA-FLS [84]. Furthermore, Klosech et al. systemically investigated the apoptotic effects of Curcumin on MH7A cells and its related molecular mechanisms, and the results showed that Curcumin has notable pro-apoptotic effects on MH7A via modulation of the NF-κB and MAPK signal pathways [85]. In 2017, Fang et al. studied the effects of Celastrol (**9**) on activated FLS from RA patients. The results showed that Celastrol attenuated the proliferation of RA-FLS, and inhibited phosphorylation of IKK and IκBα as well as down-regulated NF-κB p65 [86]. The monomer of 1,7-Dihydroxy-3,4-dimethoxyxanthone (XAN, **20**) is isolated from *Securidaca inappendiculata* Hassk, and can induce apoptosis in RA-FLS by inhibiting the activities of NF-κB and p38. It is worth noting that XAN can also regulate the transcription of X-linked inhibitor of apoptosis protein (XIAP), mediate the occurrence of Caspase cascade reaction, and induce cell apoptosis [87,88]. Furthermore, previous research in 2017 also found that *Jinwu-Jiangu* decoction (JJD) could reduce inflammatory symptoms of CIA rats and inhibit tumor like hyperplasia of FLS, and the in-depth study found that JJD medicated serum could promote FLS apoptosis by interfering with the expressions of NF-κB p65, IKK-α, and IKK-β [89,90]. Another paper by Wang et al., in 2017, reported that Baicalin (**21**) (10, 20, and 30 μM) could inhibit the proliferation of RA-FLS by decrease of NF-κB p65, phospho-NF-κB p65 and acetyl-NF-κB p65, as well as pro-inflammatory cytokines [91]. Piperlongumine (PLM, **22**) is the main component of *Piperlongum Linn*. Studies have found that low-dose PLM (1 μM) can inhibit the proliferation of RA-FLS, and high-dose PLM (15 μM) can induce apoptosis in RA-FLS. Further studies have found that PLM intervention can reduce the phosphorylation of NF-κB P65, suggesting that NF-κB pathway is involved in regulating the activity and function of FLS [92]. Recently, Wang and Zhao investigated the effect of Kaempferitrin (**23**), a natural flavonoid glycoside comprehensively existed in plants, on MH7A cells. The related results suggested that Kaempferitrin (5, 10, and 20 μM) can trigger apoptosis in MH7A cells by blocking activation of NF-κB and protein kinase B (Akt)/mammalian target of rapamycin (mTOR) pathways (Akt/mTOR) [93]. In 2018, Zhang et al. reported that by photodynamic therapy, Hypericin (**24**) (0.25–4 μM) could induce NF-κB mediated apoptosis in MH7A cells via increasing ROS, cleaved Caspase-9, Cleaved PARP, whereas decreasing NF-κB p65 [94]. The α-Mangostin (**25**) is an active monomer isolated from the *Garcinia mangostana* Linn, and an interesting study by Zuo et al. reported that α-Mangostin (15 μg/mL) decreased XIAP and increased cleaved Caspase-3, as well as inhibited phosphorylation of NF-κB p65, IκB, and IKK in FLS, suggesting the pro-apoptotic potential of α-mangostin in FLS which is closely related to inhibition of NF-κB [95]. The potential mechanisms of herbal medicine for inducing apoptosis in this part are summarized in Figure 3 and Table 1.

### 4.4. MAPK Mediated Apoptotic Pathway

MAPK pathway is an important signal transduction pathway in eucaryotic cells, which exists in most cells and is regulated step by step by MAP kinases kinases kinases (MAPKKK) and mapkinases kinases (MAPKKK). In brief, extracellular signal stimulation phosphorylates receptors on cell membrane, such as Ras, binds and activates intracellular MAPKKK, such as Raf. Activated MAPKKK then activates MAPKK, and MAPKK reactivates MAPKs to transmit signals to cells and their nuclei, and this pathway playing a crucial role in the physiological process of cell proliferation, differentiation, as well as apoptosis [96,97,98]. MAPKs mainly include extracellular regulated protein kinases (ERK), P38, and c-Jun N-terminal kinase (JNK). Generally speaking, ERK regulates Bcl-2 expression and is mainly involved in cell proliferation and differentiation. JNK and p38 are mainly involved in the stress response and apoptosis of cells by regulating the expression of downstream proteins, such as Bax, GADD153, and c-Jun [99,100]. It has been reported that MAPKs also play crucial roles in activation of Caspase cascades [101,102]. Currently, effects of the MAPK pathway in immune diseases such as RA have been comprehensively investigated. An increasing number of studies have found that MAPKs expressions in synovial tissues of RA patients are abnormally increased, which promotes synovial proliferation. Consequently, MAPKs are considered as promising potential treatment targets for treating RA [103,104,105,106].

In recent years, the effects of herbal medicine and its active monomers on MAPK pathway in articular synovium have been comprehensively reported in vivo and in vitro. In 2007, Liagreh et al. found that Diosgenin (**26**) can induce apoptosis in RA-FLS cells, and Diosgenin (40 μM) can activate p38 and JNK, but inhibit phosphorylation of ERK. In addition, DNA fragmentation induced by Diosgenin could be reduced by SB203580 and SP600125 (p38 and JNK inhibitors) [107]. Another similar study also reported that Hecogenin (**27**) and Tigogenin (**28**), which are similar to Diosgenin (**26**) in structure, have anti-proliferation and pro-apoptotic potential in FLS of arthritis patients cultured in vitro, but these two saponins only seem to activate p38, and have no significant effect on the phosphorylation of JNK and ERK [108]. Later, in 2009, Shin et al. studied the effects of Apigenin (**29**) which is a dietary plant-flavonoid and known natural compound with various bioactivities on MH7A cells. Form a systemic research in vitro, the authors found that Apigenin (25, 50, and 100 μM) intervention can significantly induce the apoptosis of RA-FLS by activating ERK1/2 and activating Caspases-3 and -7 [109]. In addition, Zuo et al. reported that XAN (**20**) (10, 30 μg/mL) can induce apoptosis and cell cycle arrest in MH7A cells which can be blocked by MAPKs inhibitors, and XAN intervention can increase the proportion of Bax/Bcl-2 in MH7A cell, as well as up-regulate the phosphorylation of ERK, JNK, and p38 [110]. β-Elemene (**30**) is a known natural sesquiterpene compound, treatment with β-Elemene (10–200 μg/mL) can improve the phosphorylation level of p38 and promote cell apoptosis in RA-FLS, and p38 inhibitors can significantly reverse the pro-apoptotic effect of β-Elemene, consequently these results suggested that the MAPK pathway is closely involved in the pro-apoptotic effects of β-Elemene on RA-FLS [111].

**Figure 4:**
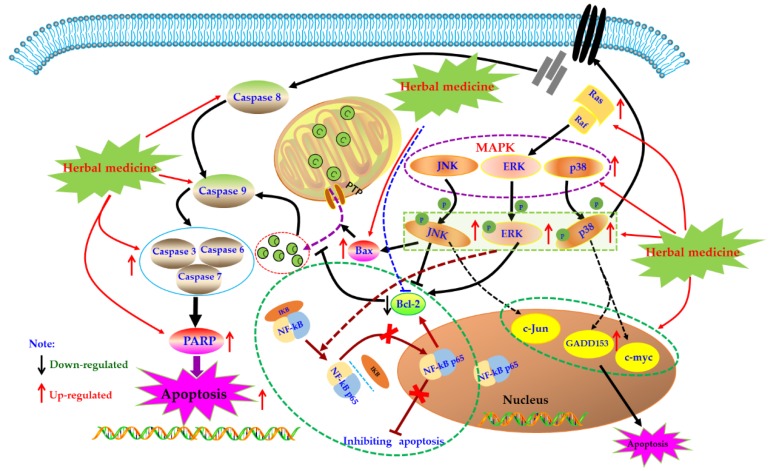
Mitogen-activated protein kinase (MAPK) mediated apoptotic pathway in FLS induced by herbal medicines.

However, there are also some investigations that showed that suppression of the MAPK signal pathway might be beneficial for induction of apoptosis in FLS. Triptolide (**31**) is a known natural monomer isolated from the *Tripterygium wilfordii* with immunosuppressive and anti-inflammatory activities. It is reported that Triptolide (0.28–140 nM) possessed notable anti-proliferative effects on RA-FLS, as well as induced cell cycle arrest and apoptosis in RA-FLS. Furthermore, the possible mechanism is related to suppression of Ras-MAPK signaling [100,112]. Brucine (**32**) is an important alkaloid of Strychni Semen, and is reported by Tang that Brucine (0.125–2 mg/mL) has potential inhibitory effect on proliferation of TNF-αstimulated RA-FLS cells via down-regulating JNK MAPK and p-JNK MAPK [113]. Previous literatures have showed that the MAPK signal could activate the NF-κB signal which is also a known anti-apoptosis signal pathway in FLS cells [114], consequently we speculated that in a certain condition, blocking MAPK signal might also induce cell proliferation suppression or apoptosis in FLS. The potential mechanisms of herbal medicine for inducing apoptosis in this part are summarized in Figure 4 and Table 1.

### 4.5. ERS Mediated Apoptotic Pathway

As an important organelle of the cell, the endoplasmic reticulum (ER) can guide the synthesis, folding, and secretion of proteins in eukaryotic cells. The endoplasmic reticulum stress (ERS) of cells caused by endoplasmic reticulum dysfunction can enhance protein folding ability, retards translation of most proteins, as well as accelerate protein degradation, etc., which is the self-protective mechanism of cells [115]. When ERS function is abnormal, Caspase-12 can be activated to directly induce apoptosis. It can also activate the unfolded protein response (UPR), induce the expression of molecular chaperones such as glucose-2 regulated protein 78kD (GRP78), GRP94, Bip, etc., regulate the Irel/xBPT pathway, p-ERK/eIF2 α pathway, and up-regulate the expression of CCAAT/enhancer binding protein epsilon (CHOP), indirectly promoting apoptosis. In addition, ESR can induce the opening of calcium channels, promote Ca^2+^ outflow, and break the Bax/bcl-2 balance. These responses are collectively called endoplasmic reticulum associated with death (ERAD) [116,117,118].

**Figure 5:**
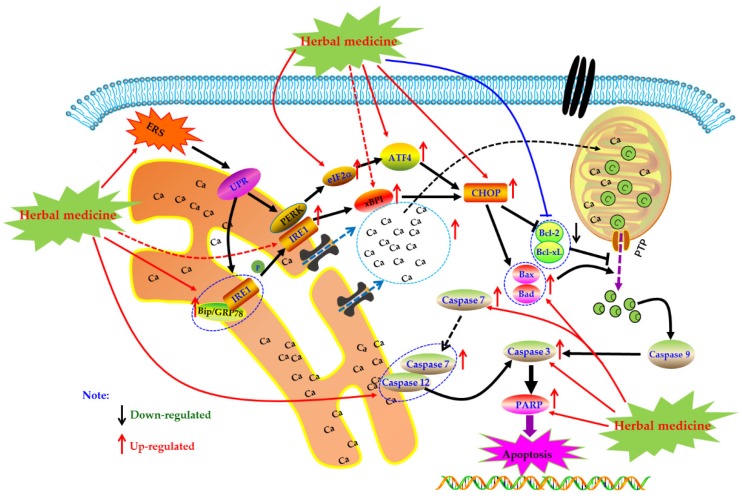
Endoplasmic reticulum (ER) mediated apoptotic pathway in FLS induced by herbal medicines.

Currently, it is abundantly reported that herbal medicines and its monomers can induce apoptosis of FLS through ERS pathway to exert anti-RA activity. In 2014, Jeong et al. found that Hempseed oil (0–2.5%) can reduce the survival rate of MH7A cells via promoting apoptosis in MH7A with a time-, dose-dependent manner. Further mechanism studies of the pro-apoptotic activities of Hempseed oil suggested that Hempseed oil can increase CHOP expression in MH7A cell, which is an important transcription factor closely related to apoptotic effect of Hempseed oil in MH7A cells [119]. Later, in 2015, Kim et al. investigated the effects of a novel chalcone derivative named (E)-3-(3,5-dimethoxyphenyl)-1-(1-hydroxynaphthalen-2-yl)-prop-2-en-1-one (DK-59, **33**) on MH7A cells. The results showed that DK-59 reduced cell viability and induced ROS production and apoptosis in MH7A cells via up-regulating ATF4 and CHOP, activating Caspase-7 and PARP, as well as increasing phosphorylation of eIF2α [120]. Similarly, the extracts of *Eupatorium japonicum* Thunb (EJTE, 37.5 μg/mL) also showed apoptosis inducing activities in RA-FLS via ERS-mediated apoptotic pathway, such as up-regulation of ATF4 and CHOP [121]. Another in vitro experiment by Lu et al. showed that in the presence of H_2_O_2_, resveratrol (**1**) (50–400 μM) could induce the apoptosis in FLS with a dose-dependent manner. Meanwhile, resveratrol treatment increased the expression of Caspase-12 and CHOP, suggesting that ERS was involved in the occurrence of FLS apoptosis. Additionally and interestingly, resveratrol did not seem to cause intracellular calcium overload [122]. The potential mechanisms of herbal medicine for inducing apoptosis in this part are summarized in Figure 5 and Table 1.

### 4.6. PI3K-Akt Mediated Apoptotic Pathway

Phosphoinositide 3 kinase (PI3K) is an important kinase, when phosphorylated, activates inositol and phosphatidylinositol, catalyzing Akt migration to the membrane and complete activation. Activated Akt is involved in physiological activities such as cell survival, growth, proliferation, cell migration, and angiogenesis by activating a series of downstream intracellular proteins. However, the abnormal regulation of the PI3K-Akt pathway may lead to the increase of these activities and induce serious diseases in the body. Studies have found that the PI3K-Akt pathway is abnormally activated in synovial cells of RA patients, which can also lead to increased expression of anti-apoptotic genes in cells [123,124]. More and more studies have proved that inhibiting the abnormally activated PI3K-Akt pathway can induce RA-HFLS apoptosis, and improve synovial hyperplasia, cartilage destruction, and other pathological phenomena of RA patients [125,126]. Currently, increasing evidence has revealed that regulation of PI3K-Akt pathway is also an important way for herbal medicines to treating RA.

#### 4.6.1. Herbal Medicine Extracts

In 2012, an in vivo experiment showed that the apoptosis rate of rat synovial fibroblasts induced by IL-1β increased by intervention of total saponin of *Dioscoreae Nipponicae* Rhizoma (TSDN, 100 μg/L), and at the same time, the phosphorylation levels of PI3K and Akt protein in FLS cells were down-regulated, suggesting that PI3K-AKT pathway was involved in the apoptotic activity of TSDN [127]. Recently, Pan et al. reported that the intervention of *Duan tengyimu* decoction (DTYD, 100 and 200 μg/mL) significantly inhibited the activation of PI3K and Akt, further enhanced the expression of Bax, and reduced the expression of Bcl-2 in RA-FLS [128]. In addition, it is reported that medicated serum of *Shuangwuxuan bi* granule also showed similar pro-apoptotic effect on RA-FLS, and the mechanism was related to the exception of PI3K/Akt signal pathway [129]. *Heiguteng-Zuifenghuoluo* Capsule (HGTZFC) is a Chinese patent medicine used for treating RA, Liu et al. reported that HGTZFC (0.315 g/kg) could down-regulate the HIF-α, p-PI3K, p-Akt, and Bcl-2, whereas up-regulate Bax in synovial tissue of CIA rats [130].

#### 4.6.2. Monomers from Herbal Medicine

**Figure 6:**
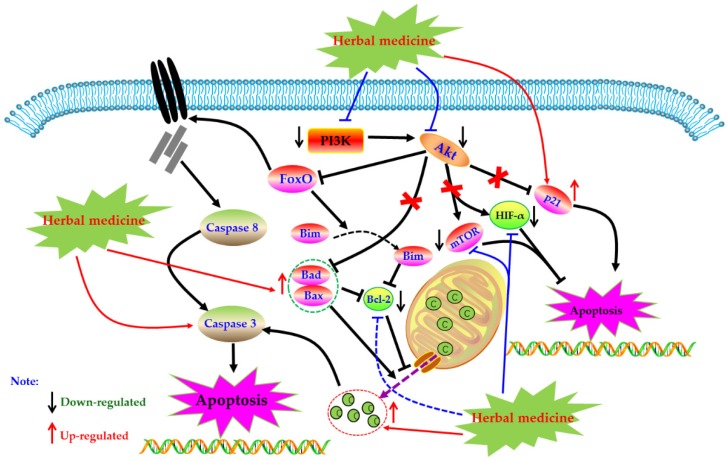
PI3K-Akt mediated apoptotic pathway in FLS induced by herbal medicines.

In 2011, Zhang et al. found that Genistein (**34**) (50, 100, and 200 μM) can induce apoptosis in vitro cultured FLS of CIA rats, and the mechanism regulates the expression of apoptosis-related proteins Bax and Bcl-2 by inhibiting Akt expression [131]. Another study by Li et al. reported that Tanshinone IIA (**12**, 40 µM) can promote apoptosis in RA-FLS via up-regulating lncRNA GAS5, cleaved Caspase-3 and -9 and inhibiting PI3K/Akt signaling [132]. In addition, Yang studied the effect of Anacardic acid (**35**) on RA, and found that anacardic acid has good therapeutic effect on symptoms of CIA rats, and the Anacardic acid (5, 30, and 60 µM) can induce apoptosis of the RA-FLS, and the possible mechanism is closely related to the PI3K/Akt signal pathway [133]. Another study in 2019 by Zuo and Wang reported that Juglone (30 µM) can promote the apoptosis in RA-FLS via inhibiting the phosphorylation of Akt and increasing expression of p21 [134]. Besides, from the results of Feng et al., Diosgenin (**26**) (10, 20, and 40 μg/mL) could also result in apoptosis in RA-FLS via regulation of the proteins related to PI3K-Akt signal [135]. In a recent study, Wang et al. investigated the effect of Pectolinarin (**36**) (20 µM) on RA-FLS, and found that besides mitochondrial dependent apoptotic pathway, regulation of PI3K/Akt signaling might be also an important molecular mechanism responding to the apoptosis inducing effects of Pectolinarin [136]. The potential mechanisms of herbal medicine for inducing apoptosis in this part are summarized in Figure 6 and Table 1.

### 4.7. Other Reported Pathways

Additionally to the apoptotic pathways mentioned above, other mechanisms also reported to be closely related to the apoptosis inducing activities of herbal medicines on FLS. Previous research has also revealed that janus kinase/signal transducers and activators of transcription (JAK-STAT) signal is closely correlated to the apoptosis induced by herbal medicines. Ren et al. reported that TAHP (**6**) (50 and 250 μM) treatment induced apoptosis in FLS, and can down-regulate Jak2 and STAT3, resulting in inhibition of the Jak2/STAT3 signal pathway [60]. Another report by Yang et al. also revealed that the pro-apoptotic effect of Matrine (**37**) (0.75 mg/mL) which is a known natural monomer from *Sophora flavescens* on rFLS in CIA rats was achieved by inhibiting the JAK/STAT pathway [137]. Recently, Zhang et al. suggested that suppression of JAK-STAT signaling is also an important molecular mechanism for the pro-apoptotic effects of GZSD in MH7A cells [16]. In 2013, Celastrol (**9**) (1–5 µM) was reported to be an active neutral compound that could dose-dependently induce apoptosis of RA-FLS, the possible mechanism is the induction of DNA damage, the G2/M phase of the cell cycle arrest and apoptosis related proteins (Bax/Bcl-2, Caspase-3 and -9) [138]. In 2016, Ren studied the inhibitory effect of 10-Hydroxycamptothecine (10-HCPT) (**38**) on proliferation of RA-FLS, and the results showed that 10-HCPT (1 and 10 μg/mL) induced obvious apoptosis in RA-FLS. In addition, the further investigation revealed that the pro-apoptotic effects of this compound might be related to down-regulation of VEGF and MMP-3 [139]. In 2017, Yao et al. reported that three natural compounds including Tamaractam (**39**), *Cis*-N-feruloyl-3-O-methylaids **(40**), and *Trans*-N-feruloyl-3-*O*-methylaids (**41**) isolated from *Tamarix ramosissima* showed in vitro pro-apoptotic effects on RA-FLS and up-regulated caspase-3 and-7. Flow cytometry results showed these compounds can increase the sub-G1 fraction in the cell cycle, suggesting that activation of the Caspase family and induction of cycle arrest are involved in pro-apoptotic effects [140]. Later, in 2018, it was also reported that *Huangqichongteng* Drink (HQCD) can induce the synovial cells in G0/G1 stage of cell growth, promoting apoptosis in RA-FLS [141]. Besides, it is also found resveratrol (**9**) (40–320 µM) down-regulated the autophagy related proteins (LC3A/B and ATG-5) in H_2_O_2_ induced FLS cells, and increase the ROS production and Ca^2+^ release, resulting in apoptosis of FLS [142]. Besides, it is reported that Wogonin (**42**), total glucosides of paeonia (TGP), Cinnamic aldehyde (CINA, **43**), and Paclitaxel (**44)** also have pro-apoptosis effects on the RA-FLS [143,144,145,146].

**Figure 7:**
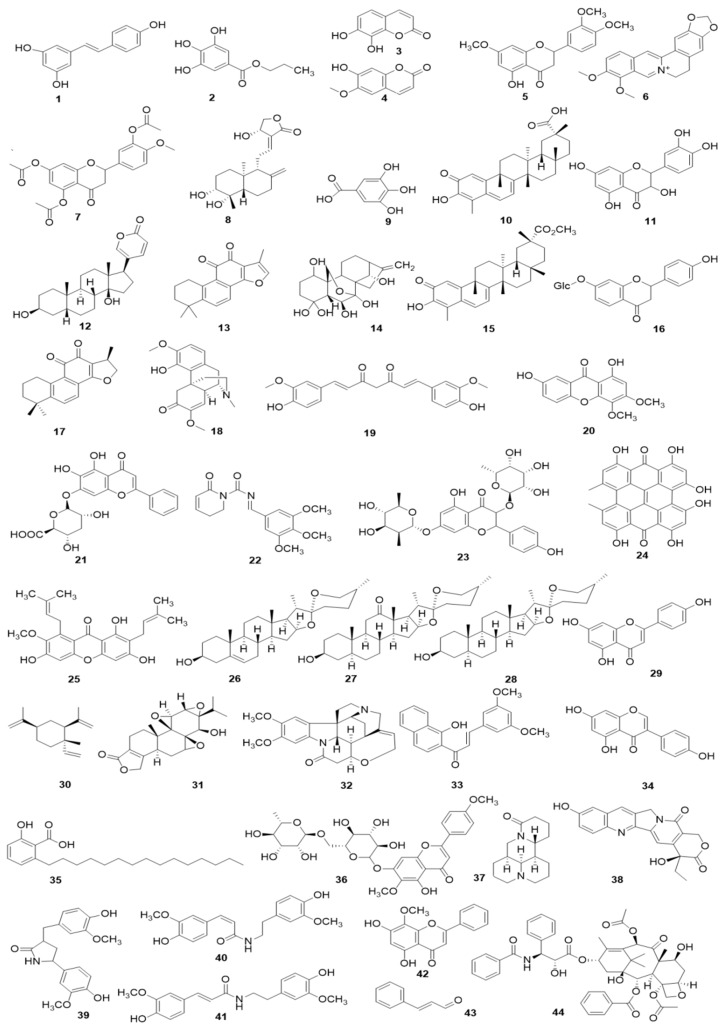
The related monomers for inducing apoptosis in FLS. Resveratrol (**1**), Propyl gallate (**2**), Daphnetin (**3**), Scopoletin (**4**), 7, 3′-dimethoxy hesperetin (**5**), Berberine (**6**), 5,7,3′-triacetyl hesperetin (TAHP, **7**), Andrographolide (**8**), Gallic acid (**9**), Celastrol (**10**), Quercetin (**11**), Bufalin (**12**), Tanshinone IIA (**13**), Oridonin (**14**), Pristimerin (**15**), Liquiritin (**16**), Cryptotanshinone (**17**), Sinomenine (**18**), Curcumin (**19**), 1,7-dihydroxy-3,4-dimethoxyxanthone (XAN, **20**), Baicalin (**21**), Piperlongumine (**22**), Kaempferitrin (**23**), Hypericin (24), α-mangostin (**25**), Diosgenin (**26**), Hecogenin (**27**), Tigogenin (**28**), Apigenin (**29**), β-Elemene (**30**), Triptolide (**31**), Brucine (**32**), (E)-3-(3,5-dimethoxyphenyl)-1-(1-hydroxynaphthalen-2-yl)-prop-2-en-1-one (DK-59, **33**), Genistein (**34**), Anacardic acid (**35**), Pectolinarin (**36**), Matrine (**37**), 10-Hydroxycamptothecine (10-HCPT, **38**), Tamaractam (**39**), Cis-N-feruloyl-3-O-methylaids (**40**), Trans-N-feruloyl-3-O-methylaids (**41**), Wogonin (**42**), Cinnamic aldehyde (CINA, **43**), and Paclitaxel (**44**).

**Figure 8:**
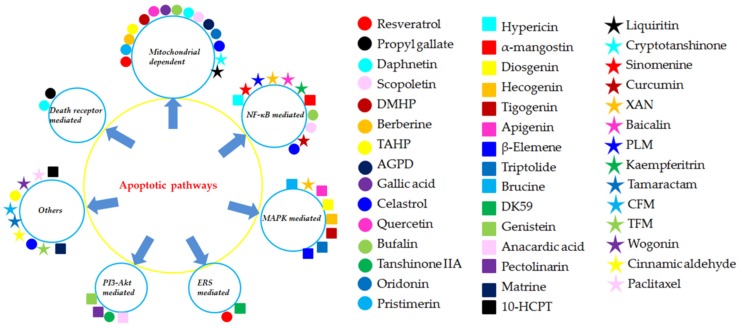
The different apoptotic pathways of the natural monomers isolated from herbal medicines. 10-HCPT, 10-Hydroxycamptothecine; AGPD, Andrographolide; CFM, *cis*-n-feruloyl-3-o-methylaids; CINA, Cinnamic aldehyde; DK-59, (E)-3-(3,5-dimethoxyphenyl)-1-(1-hydroxynaphthalen-2-yl)prop-2-en-1-one; DMHP, 7,3′-Dimethoxy Hesperetin; PLM, Piperlongumine; TAHP, 5,7,3′-triacetyl-hesperetin; TFM, *trans*-n-feruloyl-3-*O*-methyla; XAN, 1,7-dihydroxy-,4-dimethoxyxanthone.

The related monomers in the section (*Effect of FLS apoptosis in RA*) for inducing apoptosis in FLS and its possible molecular mechanism are summarized in Table 1 and Table 2 and Figure 7 and Figure 8.

## 5. Conclusions and Perspectives

It is reported that after simulation by the pro-inflammatory cytokines such as TNF-α, the fibroblast-like synoviocytes (FLS) would grow rapidly like tumor cells, and development of RA is closely related to an imbalance between cell proliferation, survival, and death of FLS [16,147]. Thus, promoting death of FLS in RA patients might be a feasible way for treating RA, and compared with other therapeutic approaches, targeting FLS might ameliorate clinical symptoms of RA without suppressing systemic immunity. Apoptosis is the main typical programmed cell death ways (PCD) and physiological cell suicide processes. Recently, apoptosis is considered as an ideal way for treatment of cancers, and increasing studies have also indicated that inducing apoptosis in FLS of RA patients would be beneficial for controlling the symptoms and development of RA [16,18].

It is generally recognized that herbal medicines have played important roles in human health maintenance and disease therapy for thousands of years before the appearance of synthetic drugs [148]. Currently, natural products including extracts and monomers derived from herbal medicines are receiving increasing attention in the world, and are commonly considered as precious resource for screening and finding novel candidate drugs with less toxicity [14,149]. In addition, a large number of herbal medicines and its components have the pro-apoptotic activities on FLS, and we have summarized these herbal medicines in this present review. Taken together, apoptosis induction of FLS is an important molecular mechanism for herbal medicine and its active components in the treatment of rheumatoid arthritis, and the main related signal pathways are concluded as death receptors mediated apoptotic pathway, mitochondrial dependent apoptotic pathway, NF-κB mediated apoptotic pathways, MAPK mediated apoptotic pathway, ERS mediated apoptotic pathway, PI3K-Akt mediated apoptotic pathway, and other reported pathways such as JAK-STAT signal pathway. Understanding the apoptosis induction pathways in FLS of these natural medicines will not only help clear molecular mechanisms of herbal medicines for treating RA but also be beneficial for finding novel candidate therapeutic drugs from natural herbal medicines. Consequently, we expect the present review will highlight the importance of herbal medicines and its components for treating RA via induction of apoptosis in FLS, and provide some directions for the future development of these mentioned herbal medicines as anti-RA drugs in clinical.

## Figures and Tables

**Table 1:** Apoptosis inducting activities of herbal medicine and its active components in fibroblast-like synoviocytes.

Potential Pathways	Detail Mechanisms	Extracts/Monomers (dose/concentration)	Cells/Tissues	Related Genes/Proteins	Reference
Death receptors mediated apoptotic pathway	Up-regulating Fas and FasL; Down-regulating Bcl-2	XFC (1.8 g/kg, p.o.)	Synovia tissues in rats	Fas, FasL, Bcl-2	[40]
	Up-regulating Fas	ERSM (0.4 mg/mL)	RA-FLS	Fas	[41]
	Up-regulating Caspase-8, Fas and FasL	FSBT (9.5–38 g/kg, p.o.)	Synovia tissues in rats	Caspase-8, Fas, FasL	[42]
	Up-regulating Caspase-8 and FLIP	Resveratrol (50–400 μM)	rFLS (CIA)	Caspase-8, FLIP	[44]
	Increasing Fas mRNA	Propyl gallate (64 μg/mL)	RA-FLS	Fas	[48,49]
	Up-regulating Caspase -3, -8, -9, and FasL	Daphnetin (40 μg/mL)	rFLS (CIA)	FasL, TNF, Bid, Bax, Bcl-2, Cyt-C, Caspase-3, -8, and -9	[50,51]
Mitochondrial apoptotic pathway	Up-regulating Bax and Caspase-3; Down-regulating Bcl-2	SWPD (15–25 mg/mL)	RA-FLS	Bcl-2, Bax, Caspase-3	[53]
	Down-regulating Bcl-2; Activating Caspase-3; Increasing Cyt-C release	FSNC (0.33–1.32 g/kg)	rFLS (CIA)	Bcl-2, Caspase-3, Cyt-C	[54]
	Up-regulating Bax and Caspase-3; Down-regulating Bcl-2	DHJS medicated serum, (0.75, 1.5 and 3 g/kg)	rFLS (AIA)	Bax, Bcl-2, and Caspase-3	[55]
	Up-regulating p53, Bax and Caspase-3; Downreglulating Bcl-2	SDP (5–15 mg/mL)	rFLS (AIA)	p53, Bax, Caspase-3, Bcl-2	[56]
	Up-regulating p53, Bak, Cyt-C, Bax, Caspase-3, -9; Down-regulating Bcl-2 and Bcl-xL; Activating Caspase-3, -9	PHSE (25–100 μg/mL)	MH7A	p53, Bax, Bak, Bcl-2, Bcl-xL, Cyt-C, Caspase-3, -9	[57]
	Inhibiting proinflammatory cytokines; Up-regulating Caspase-3, -9 and Bax; Down-regulating Bcl-2,	GSZD (0.4–1.6 mg/mL)	MH7A, stimulated by TNF-α (20 ng/mL)	Caspase-3, -9, Bax, Bcl-2	[16]
	Up-regulating Bax; Down-regulating Bcl-2; Decreasing MCMP (Δ*ψ*m); Activating Caspase -3	Scopoletin (250–1000 μM)	rFLS (AIA), stimulated by LPS (5 μg/mL)	MCMP, Caspase-3, Bax, Bcl	[58]
	Up-regulating Caspase-3 and Bax; Down-regulating Bcl-2; Activating Caspase-3	DMHP (10–250 μM)	rFLS (AIA)	Caspase-3, Bax, Bcl-2	[59]
	Increasing Caspase-3, -9, Bax and PARP; Decreasing Bcl-2, Bcl-xL and MCMP (Δψm)	Berberine (5–75 μM)	RA-FLS	Caspase-3, -9, Bax, PARP, Bcl-2, Bcl-xL	[60]
	Up-regulating Bax and Caspase-3; Down-regulating Bcl-2	TAHP (50, 250 μM)	rFLS (AIA)	Bcl-2, Bax, Caspase-3	[61]
	Up-regulating Bax and Cyt-C; Down-regulating Bcl-2; Activating Caspase -3	AGPD (10–30 μM)	RA-FLS	Bax, Bcl-2, Caspase-3, Cyt-C	[62]
	Increasing Caspase-3 activity; Up-regulating Bax and p53; Down-regulating Bcl-2	Gallic acid (0.1, 1 μM)	RA-FLS	Caspase-3,Bax, p53, Bcl-2	[63]
	Increasing Caspase-3, -9, PARP, Fas and Bax; Decreasing Bcl-2 and MCMP (Δψm)	Celastrol (1, 2, 5 μM)	RA-FLS	Caspase-3, -9, PARP, Fas, Bax, Bcl-2	[64]
	Increasing Caspase-3, -9, Cyto C; Decreasing Bcl-2	Quercetin (100–300 μM)	RA-FLS	Caspase-3, -9, Cyto C, Bcl-2	[65]
	Up-regulating Bax; Down-regulating Bcl-2; Activating Caspase -3 and PARP Increasing Cyt-C release; Decreasing MCMP (Δ*ψ*m)	Bufalin (10–40 nM)	RA-FLS, stimulated by IL-1β (1 ng/mL)	MCMP, Bax, Bcl-2, PARP, Caspase-3, Cyt-C	[66]
	Up-regulating Bax, cytosolic Cyto C, Apaf-1, Caspase-3, -9; Down-regulating Bcl-2	Tanshinone IIA (2.5–20 μM)	RA-FLS	Bax, Cyto C, Apaf-1, Caspase-3, -9, Bcl-2	[67]
	Up-regulating Caspase-3,-8, -9, Bax, Bid and Cyt-C; Down-regulating Bcl-2	Daphnetin (40 μg/mL)	rFLS (CIA)	FasL, TNF, Cyt-C, Bid, Bax, Bcl-2, Caspase-3, -8, -9	[68]
	Decreasing MCMP (Δ*ψ*m); Increasing Cyt-C release; Up-regulating Caspase-3 -9 and PARP	Oridonin (5–40 μM)	RA-FLS, stimulated by IL-1β (1 μg/mL)	MCMP, Caspase-3,-9, PARP, Cyt- C	[69]
	Down-regulating MCMP (Δ*ψ*m)	Resveratrol (50–400 μM)	rFLS (AIA), stimulated by H_2_O_2_ (5 μM)	MCMP	[70,71]
	Up-regulating Bax and Caspase-3; Down-regulating Bcl-2	Pristimerin (0.75–3 μM)	rFLS (AIA)	Bax, Caspase-3, Bcl-2	[73]
	Down-regulating Bcl-2/Bax	Liquirtin (0.345–34.5 μM)	RA-FLS	Bcl-2, Bax	[74]
	Increasing ROS; Up-regulating Bad, Caspase-3, PARP; Down-regulating Bcl-2	Cryptotanshinone (5 μM)	MH7A cells and RA-FLS cells	Bcl-2, Bad, Caspase-3, PARP	[75]
NF-κB mediated apoptotic pathway	Down-regulating Bcl-2, MyD88 and TRAF-6	Sinomenine (0.5–3.2 mM)	RA-FLS	MyD88, TRAF-6, Bcl-2	[81,82,83]
	Up-regulating Bax and IκBα; Down-regulating Bcl-2, p-IKK and p-IκBα	Scopoletin (250–1000 μM)	rFLS (AIA), stimulated by LPS (5 μg/mL)	Bax, IκBα, Bcl-2, p-IKK, p-IκBα	[57]
	Inhibiting phosphorylation of NF-κB and IκBα; Activating Caspase-3, -7	Curcumin (12.5–80 μM)	RA-FLS, MH7A, stimulated by IL-1β (10 ng/mL)	NF-κB, IKBα, Caspase-3, -7	[84,85]
	Inhibiting phosphorylation of IKK and IκBα; Down-regulating NF-κB p65	Celastrol (0.25–2 μM)	RA-FLS	IKK, IκBα, NF-κBp65	[86]
	Inhibiting phosphorylation of NF-κBp65 and IKKβ, IκB and MSK1; Down-regulating XIAP and Cyclin D1; Up-regulating GADD45a	XAN (8.7–34.7 μM)	RA-FLS	NF-κBp65, IKKβ, IκB, MSK1, XIAP, Cyclin D1, GADD45a	[87,88]
	Down-regulating NF-κB p65, IKKα and IKKβ	JJD medicated serum	RA-FLS	NF-κBp65, IKKα, IKKβ	[89,90]
	Decreasing NF-κB p65, phospho-NF-κB p65 and acetyl-NF-κB p65, as well as pro-inflammatory cytokines	Baicalin (10, 20, 30 μM)	RA-FLS	NF-κB p65	[91]
	Inhibiting phosphorylation of NF-κBp65	PLM (5–20 μM)	RA-FLS, stimulated by TNF-α (10 ng/mL)	NF-κBp65	[92]
	Down-regulating p-NF-κBp65 and p-IκB	Kaempferitrin (5–20 μM)	MH7A	NF-κBp65, p-NF-κBp65, IκB, p-IκB,	[93]
	Increasing ROS, cleaved Caspase-9, Cleaved PARP; Decreasing NF-κB p65	Hypericin (0.25–4 μM)	MH7A	Caspase-9, PARP, NF-κB p65	[94]
	Down-regulating XIAP; Up-regulating Caspase 3; Inhibit phosphorylation of NF-κBp65, IκB and IKK	α-Mangostin (6–14 μg/mL)	RA-FLS, stimulated by TNF-α (10 ng/mL)	XIAP, Caspase 3, p65, IκB, IKK	[95]
MAPK mediated apoptotic pathway	Up-regulating JNK, p38; Down-regulating ERK; Increasing DNA fragmentation	Diosgenin (40 μM)	RA-FLS, stimulated by IL-1β (1 ng/mL)	JNK, ERK, p38	[107]
	Up-regulating JNK, p38α; Down-regulating ERK; Activating Caspase -3, -8, -9	Hecogenin and Tigogenin (10, 40 μM)	RA-FLS	JNK, ERK, p38α, Caspase-3,-8,-9	[108]
	Up-regulating JNK, p38α; Down-regulating ERK1/2; Activating Caspase -3, - 7 and PARP-1	Apigenin (25–100 μM)	MH7A	JNK, ERK, p38, Caspase-3, - 7, PARP-1	[109]
	Inhibiting Ras-MAPK signaling	Triptolide (0.28–200 nM)	RA-FLS, stimulated by TNF-α (10 ng/mL)	Ras, p38, ERK, JNK	[100,112]
	Up-regulating Bax, ERK, P38 and p21; Down-regulating Bcl-2, JNK	XAN (10, 30 μg/mL)	MH7A, stimulated by TNF-α (10 ng/mL)	Bax, ERK, p38, Bcl-2, JNK	[110]
	Down-regulating JNK and p-JNK	Brucine (0.125–2 mg/mL)	RA-FLS, stimulated by TNF-α (10 ng/mL)	JNK, p-JNK	[113]
	Up-regulating p38; Activating Caspase-3, -9	β-Elemene (10–200 μg/mL)	RA-FLS	p38, Caspase- 3, - 9	[114]
ERS mediated apoptotic pathway	Up-regulating CHOP, GRP94 and GRP78; Activating PARP	Hempseed oil (2.5%)	MH7A	CHOP, PARP	[119]
	Up-regulating ATF4, CHOP and XBPI; Activating Caspase-3, -7 and PARP; Increasing phosphorylation of eIF2α, IRE1α and BiP	DK-59 (10 μM)	MH7A	ATF4, CHOP, XBPI, Caspase-3, -7, PARP, eIF2α, IRE1α, BiP	[120]
	Up-regulating ATF4 and CHOP; Activating Caspase -7 and PARP; Increasing phosphorylation of eIF2α	EJTE (37.5 μg/mL)	MH7A, stimulated by TNF-α (10 ng/mL)	ATF4, CHOP, Caspase- 7, PARP, IeIF2α	[121]
	Up-regulating Bax and CHOP; Down-regulating Bcl-2	Resveratrol (50–400 μM)	rFLS (AIA), stimulated by H_2_O_2_ (5 μM)	CHOP, Bcl-2	[122]
PI3K/AKT mediated apoptotic pathway	Up-regulating Bax; Down-regulating Bcl-2; Inhibiting phosphorylation of PI3K and Akt	DTYD (100, 200 μg/mL)	RA-FLS	Bax, Bcl-2, PI3K, Akt	[128]
	Up-regulating Bax; Down-regulating Bcl-2, PI3K and Akt	SWXB medicated serum (4.32, 8.64, 17.28 g/kg)	RA-FLS	Bax, Bcl-2, PI3K, Akt	[129]
	Inhibiting phosphorylation of PI3K and Akt	TSDNR (100 μg/L)	rFLS, stimulated by IL-1β (10 μg/L)	PI3K, Akt	[127]
	Down-regulating HIF-α, p-PI3K, p-Akt, Bax, Bcl-2 in synovial tissue of CIA rats	HGTZFC (0.315 g/kg)	Synovial tissue of CIA rats	HIF-α, p-PI3K, p-Akt, Bcl-2, Bax	[130]
	Up-regulating Bax; Down-regulating Bcl-2; Inhibiting phosphorylation of Akt	Genistein (50–200 μM)	rFLS (CIA)	Bax, Bcl-2, Akt	[131]
	Up-regulating lncRNA GAS5; Up-regulating cleaved Caspase-3, -9; Inhibiting PI3K/Akt signaling	Tanshinone IIA (40 µM)	RA-FLS	lncRNA GAS5, Caspase-3, -9, PI3K, Akt	[132]
	Decreasing Akt and miR-633	Anacardic acid (5, 30 and 60 µM)	RA-FLS, stimulated by TNF-α (10 ng/mL)	Akt	[133]
	Inhibiting the phosphorylation of Akt; Increasing p21	Juglone (30 µM)	RA-FLS	Akt, p21	[134]
	Up-regulating cleaved Caspase-3, Bax; Down-regulating PI3K, Akt, mTOR	Diosgenin (10, 20, 40 μg/mL)	RA-FLS	Caspase-3, Bax, PI3K, Akt, mTOR	[135]
	Up-regulating Bax; Down-regulating Bcl-2, PI3K and Akt	Pectolinarin (10, 20 µM)	RA-FLS	Bax, Bcl-2, PI3K, Akt	[136]
Other	Down-regulating Jak2 and STAT3; Inhibiting Jak2/STAT3 signaling	TAHP (50, 250 μM)	rFLS (AIA)	Jak2, STAT3, p-STAT3	[60]
	Up-regulating Bax, LC3A, ATR, Chk-1 and ATR; Down-regulating Bcl-2, FasR and Cyclin-B1; Increasing phosphorylation of Cdc-2, -25; Activating Caspase-3, -9 and PARP; Increasing DNA damage G2/M phase of stagnation	Celastrol (1–5 µM)	RA-FLS	Bax, LC3A, ATR, Chk-1, γ-H2AX, Bcl-2, FasR, Cyclin-b1, Cdc-25, Cdc-2, Caspase-3, -9, PARP	[138]
	Inducing pro-apoptosis effects on the RA-FLS	Wogonin (111, 0–200 μM)	RA-FLS		[143]
	Up-regulating Bax and Caspase-3; Down-regulating Bcl-2; Inhibiting phosphorylation of JAK2, STAT-1, -3	Matrine (0.75 mg/mL)	rFLS (CIA)	Bax, Bcl-2, Caspase-3, JAK2, STAT1, -3	[137]
	Down-regulating VEGF and MMP-3	10-HCPT (1, 10 μg/mL)	RA-FLS	VEGF and MMP-3	[139]
	Activating Caspase-3, -7; Increasing G1 cell cycle	Tamaractam; CFM; TFM (0.1, 1 µM)	RA-FLS	Caspase-3, -7	[140]
	Inducing growth stagnation of synovial cells at G0/G1 stage	HCTD (6.25–100 µg/mL)	rFLS (CIA)		[141]
	Up-regulating Bax and LC3A; Down-regulating Bcl-2, Atg5 and LC3B; Increasing ROS production and Ca^2+^ release	Resveratrol (40–320 µM)	RA-FLS, stimulated by H_2_O_2_ (5 μM)	Bax, LC3A, Bcl-2, Atg5, LC3B	[142]
	Inducing pro-apoptosis effects on the RA-FLS	TGP (5–50 μg/mL)	RA-FLS		[144]
	Inhibiting pro-inflammatory cytokines; Down-regulating Bcl-2, Jak2, STAT-3, -5; Inhibiting Jak2/STAT3 signaling	GSZD (0.4–1.6 mg/mL)	MH7A, stimulated by TNF-α (20 ng/mL)	SOCS1, JAK2, STAT-3, -5	[16]
	Inducing pro-apoptosis effects on the RA-FLS	CINA (1,10 μg/mL)	RA-FLS		[145]
	Inducing pro-apoptosis effects on the RA-FLS	Paclitaxel (2,4,8 μM)	RA-FLS		[146]

10-HCPT, 10-Hydroxycamptothecine; AGPD, Andrographolide; AIA, adjuvant-induced arthritis; CFM, cis-n-feruloyl-3-o-methylaids; CIA, collagen-induced arthritis; CINA, cinnamic aldehyde; DHJS medicated serum, Medicated serum of *Duhuo Jisheng* decoction; DMHP, 7,3′-Dimethoxy Hesperetin; DK-59, (E)-3-(3,5-dimethoxyphenyl)- 1-(1-hydroxynaphthalen-2-yl)prop-2-en-1-one; DTYD, *Duanteng Yimu* Decoction; EJTE, *Eupatorium japonicum* Thunb. Extracts; extracts from the roots of *Salvia miltiorrhiza* (ERSM), extracts from the roots of *Salvia miltiorrhiza;* FLIP, FLICE inhibitory protein; FSBT, *Fengshi Bitong Prescription*; FLS, fibroblast-like synoviocytes; FSNC, *Fengshining* Capsule; GSZD, *Guizhi-Shaoyao-Zhimu* decoction; HCTD, *Huangqi Chongteng* Drink; HGTZFC, *Heiguteng-Zuifenghuoluo* Capsule; JJD, *Jinwu Jiangu* Decoction; MCMP, Measurement of Mitochondrial Membrane Potentia; rFLS, FLS from rats; RA-FLS, FLS from RA patients; PHSE, Pterocarya Hupehensis Skan extracts; PLM, Piperlongumine; SDP, Polysaccharide from the Saposhnikovia divaricate; SWPD, Seaweed Polysaccharide; SWXB, *Shuang-wu-xuan-bi* granule; TAHP, 5,7,3’-triacetyl hesperetin; TFM, trans-n-feruloyl-3-*O*-methyla; TGP, total glucosides of paeonia; TSDNR, Total Saponin of Dioscoreae Nipponicae Rhizoma; XAN, 1,7-dihydroxy-, 4-dimethoxyxanthone; XFC, *Xinfeng* capsule.

**Table 2:** Monomers for inducing apoptosis in fibroblast-like synoviocytes.

Classification	Monomers	Apoptotic pathways	References
Alkaloids	Berberine	Mitochondrial dependent apoptosis	[60]
	Sinomenine	NF-κB mediated apoptosis	[81,82,83]
	PLM	NF-κB mediated apoptosis	[92]
	Brucine	MAPK mediated apoptosis	[113]
	Matrine	Inhibiting JAK/STAT	[137]
	10-HCPT	Down-regulating VEGF and MMP-3	[139]
	Tamaractam CFM TFM	Caspase activation induced apoptosis and induction of cell arrest	[140]
Flavonoids	DMHP	Mitochondrial dependent apoptosis	[59]
	TAHP	Mitochondrial dependent apoptosis Inhibiting JAK/STAT	[61]
	Quercetin	Mitochondrial dependent apoptosis	[65]
	Liquiritin	Mitochondrial dependent apoptosis	[74]
	XAN	NF-κB mediated apoptosis MAPK mediated apoptosis	[87,88] [110]
	Baicalin	NF-κB mediated apoptosis	[91]
	Kaempferitrin	NF-κB mediated apoptosis	[93]
	α-mangostin	NF-κB Mediated apoptosis	[95]
	Apigenin	MAPK mediated apoptosis	[109]
	Genistein	PI3K-Akt mediated apoptosis	[131]
	Pectolinarin	PI3K-Akt mediated apoptosis	[136]
	Wogonin	Not mentioned	[143]
Steroids	Bufalin	Mitochondrial dependent apoptosis NF-κB mediated apoptosis	[66]
	Diosgenin	MAPK mediated apoptosis	[107,108]
	Hecogenin		
	Tigogenin		
Phenylpropanoids	Daphnetin	Death receptors mediated Mitochondrial dependent apoptosis	[50,51]
	Scopoletin	Mitochondrial dependent apoptosis NF-κB mediated apoptosis	[57,58]
	Curcumin	NF-κB mediated apoptosis	[84,85]
	CINA	Not mentioned	[145]
Terpenoids	AGPD	Mitochondrial dependent apoptosis	[62]
	Oridonin	Mitochondrial dependent apoptosis;	[69]
	Celastrol	Mitochondrial dependent apoptosis; NF-κB mediated apoptosis; Inducing DNA damage and cell cycle arrest	[64] [86] [138]
	Pristimerin	Mitochondrial dependent apoptosis	[73]
	Triptolide	MAPK mediated apoptosis	[100,112]
	β-Elemene	MAPK mediated apoptosis	[114]
	Paclitaxel	Not mentioned	[146]
Quinones	Cryptotanshinone	Mitochondrial dependent apoptosis	[75]
	Hypericin	NF-κB mediated apoptosis	[94]
	Tanshinone IIA	PI3K-Akt mediated apoptosis	[132]
Others	Propyl gallate	Death receptors mediated apoptosis	[48,49]
	Gallic acid	Mitochondrial dependent apoptosis	[63]
	Resveratrol	Mitochondrial dependent apoptosis ERS mediated apoptosis	[70,71] [122]
	DK-59	ERS mediated apoptosis	[120]
	Anacardic acid	PI3K-Akt mediated apoptosis	[133]

10-HCPT, 10-Hydroxycamptothecine; AGPD, Andrographolide; CFM, cis-n-feruloyl-3-o-methylaids; CINA, Cinnamic aldehyde; DK-59, (E)-3-(3,5-dimethoxyphenyl)- 1-(1-hydroxynaphthalen-2-yl)prop-2- en-1-one; DMHP, 7,3′-Dimethoxy Hesperetin; PLM, Piperlongumine; TAHP, 5,7,3′-triacetyl hesperetin; TFM, trans-n-feruloyl-3-O -methyla; XAN, 1,7-dihydroxy-,4-dimethoxyxanthone.
